# Molecular Insights into the Interaction Between Metformin and Caffeine: The Time-Dependent Antagonism and Modulation of p53 Signaling in Cancer Cells

**DOI:** 10.3390/molecules31111877

**Published:** 2026-05-29

**Authors:** Vesna Zeljković, Mirjana Bogavac, Milan Dekić, Slaviša Minić, Elvis Mahmutović, Vanja Kunkin, Zoran Marković, Maja Karaman

**Affiliations:** 1 Department of Biomedical Sciences, State University of Novi Pazar, Vuka Karadžića 9, 36300 Novi Pazar, Serbia; vzeljkovic@np.ac.rs (V.Z.); sminic@np.ac.rs (S.M.); ehmahmutovic@np.ac.rs (E.M.); 2 Department of Obstetrics and Gynecology, Faculty of Medicine, University of Novi Sad, Hajduk Veljkova 3, 21000 Novi Sad, Serbia; mirjana.bogavac@mf.uns.ac.rs; 3 Department of Sciences and Mathematics, State University of Novi Pazar, Vuka Karadžića 9, 36300 Novi Pazar, Serbia; mdekic@np.ac.rs (M.D.); zmarkovic@uni.kg.ac.rs (Z.M.); 4 Đorđe Joanović Zrenjanin General Hospital, Dr Vase Savića 5, 23000 Zrenjanin, Serbia; kunkinbre@gmail.com; 5 Department of Biology and Ecology, Faculty of Sciences, University of Novi Sad, Trg Dositeja Obradovića 2, 21000 Novi Sad, Serbia

**Keywords:** AMPK, antitumor activity, apoptosis, caffeine, cancer cell lines, Chou–Talalay method, drug repurposing, docking, metformin, molecular docking, p53

## Abstract

**Background**: Cancer remains a major global health challenge, with treatment efficacy often limited by drug resistance and adverse effects. Drug repurposing offers promising opportunities for developing novel anticancer strategies. This study evaluated the cytotoxic, antiproliferative, and pro-apoptotic effects of metformin and caffeine, administered individually and in combination, in human cancer cell lines, as well as their potential interaction mechanisms. **Methods**: Human cervical carcinoma (*HeLa*), lung adenocarcinoma (*A549*), and colorectal carcinoma (*HT29*) cell lines were treated with metformin (0.05–50 mM) and caffeine (0.5–5 mM), either alone or in combination, for 24 and 48 h. Cell viability and proliferation were assessed using Trypan Blue and sulforhodamine B (SRB) assays. Apoptosis was analyzed by Annexin V/propidium iodide flow cytometry, and p53 expression in HeLa cells was determined by ELISA. Statistical analysis was performed using a one-way ANOVA followed by Tukey’s post hoc test. **Results**: Metformin induced dose- and time-dependent cytotoxicity in all tested cell lines, with the lowest IC_50_ values observed in *HeLa* and A549 cells after 48 h (2.28 and 3.30 mM, respectively; *p* < 0.05). Caffeine showed moderate antiproliferative activity, with the strongest effects observed at 2.03 mM in *HeLa* cells and 2.01 mM in *HT29* cells (*p* < 0.05). The combined treatment produced effects that varied depending on both the cell line and exposure time. At earlier time points, transient synergistic effects were observed in certain cell lines, particularly *HeLa* cells; however, these effects were not sustained over time. With prolonged exposure, the interaction shifted predominantly toward antagonistic effects, indicating the reduced overall efficacy of the combination compared with the expected additive outcomes. Increased apoptosis and elevated p53 expression further supported the activation of tumor-suppressive pathways. **Conclusions**: Metformin exhibited significant anticancer activity in vitro, supporting its potential repurposing in oncology. However, the addition of caffeine did not uniformly enhance its efficacy and appeared to exert context-dependent effects. Further in vivo studies are required to confirm the clinical relevance of these findings.

## 1. Introduction

Rational combinations of repurposed drugs targeting complementary cellular pathways have emerged as a promising strategy to enhance anticancer efficacy while minimizing toxicity. In this context, cancer remains a major global health challenge and one of the leading causes of mortality worldwide, highlighting the urgent need for more effective and safer therapeutic strategies [1]. Despite significant advances in oncology, conventional treatment modalities—including surgery, radiotherapy, and chemotherapy—are often limited by drug resistance, suboptimal efficacy, and considerable adverse effects [2]. These limitations highlight the urgent need for alternative therapeutic approaches, particularly those based on molecular targeting and drug repurposing strategies.

Recent epidemiological data confirm the sustained global burden of malignancies, with lung, colorectal, and cervical cancers being among the most prevalent and lethal cancer types worldwide [1,3,4]. These trends further underscore the importance of developing novel therapeutic strategies with improved safety profiles and enhanced efficacy.

In this context, drug repurposing has emerged as a promising strategy in anticancer research, offering advantages such as reduced development time, lower costs, and well-characterized pharmacokinetic and safety profiles. Among repurposed agents, metformin—a widely used antidiabetic drug—has attracted considerable attention due to its reported antiproliferative and pro-apoptotic effects in various cancer models [1,3,4]. These effects have been associated with the modulation of cellular metabolism, activation of AMP-activated protein kinase (AMPK), and regulation of tumor suppressor pathways, including p53 signaling [5].

Metformin (1,1-dimethylbiguanide hydrochloride) is a biguanide oral hypoglycemic agent and the first-line therapy for type 2 diabetes mellitus [6]. Its favorable safety profile, good tolerability, and efficacy in reducing hyperglycemia without inducing weight gain or hypoglycemia have contributed to its widespread clinical use. Metformin has gained considerable attention as a repurposed anticancer agent [7,8], with epidemiological and experimental studies demonstrating its antiproliferative and pro-apoptotic effects across multiple cancer types. However, the precise molecular mechanisms underlying these effects remain incompletely understood.

The anticancer activity of metformin involves both indirect and direct mechanisms. Indirectly, metformin reduces systemic glucose levels by suppressing hepatic gluconeogenesis, increasing insulin sensitivity, and enhancing peripheral glucose uptake through the activation of AMP-activated protein kinase (AMPK) [9]. This leads to reduced circulating insulin and insulin-like growth factor-1 (IGF-1) levels, thereby limiting tumor growth stimulation [6,9,10].

Directly, metformin exerts antiproliferative effects on tumor cells by inducing apoptosis and inhibiting protein synthesis [11,12]. It promotes cell cycle arrest in the G1 phase primarily through the activation of AMPK and inhibition of the mammalian target of rapamycin (mTOR) signaling pathway. In addition, metformin modulates autophagy, while the STK11/AMPK/mTOR axis plays a central role in regulating cellular processes, including survival, proliferation, apoptosis, and metabolism [13,14].

Caffeine is a natural methylxanthine compound with diverse biological effects, including the modulation of cellular signaling pathways relevant to cancer progression. At the molecular level, caffeine acts as a non-selective phosphodiesterase (PDE) inhibitor, leading to increased intracellular levels of cyclic adenosine monophosphate (cAMP) and cyclic guanosine monophosphate (cGMP), which are associated with the inhibition of cancer cell proliferation and induction of apoptosis [15,16].

The rationale for combining metformin and caffeine lies in their potentially complementary mechanisms of action. While metformin primarily affects cellular energy metabolism and AMPK-related signaling pathways, caffeine has been associated with the modulation of apoptosis, oxidative stress, and cell cycle regulation. Their combined application may exert additive or synergistic anticancer effects by simultaneously targeting metabolic and stress response pathways involved in tumor progression.

To the best of our knowledge, the combined effects of metformin and caffeine on p53-associated signaling pathways have not been extensively investigated using integrated in vitro and in silico approaches.

In addition, caffeine interferes with key regulators of the DNA damage response, including ataxia telangiectasia mutated (ATM) and ataxia telangiectasia and Rad3-related (ATR) kinases, thereby affecting cell cycle progression and apoptotic signaling [15,17]. Through these mechanisms, caffeine modulates cellular stress responses, influences tumor cell survival, and enhances sensitivity to metabolic stress.

Caffeine has also been reported to enhance the effects of conventional anticancer therapies, acting as a chemo- and radiosensitizer [15,18]. However, despite growing evidence regarding the individual anticancer effects of metformin and caffeine, their combined molecular interactions—particularly in relation to p53 signaling—remain insufficiently characterized. Targeting both metabolic and stress response pathways may provide a synergistic framework for enhancing anticancer efficacy [19].

In silico approaches, such as molecular docking, provide valuable insights into drug–protein interactions and may help elucidate the molecular mechanisms underlying their biological effects [20]. Recent studies have successfully applied molecular docking approaches to investigate the interactions of bioactive compounds with therapeutic protein targets in inflammatory, antioxidant, antimicrobial, and neurodegenerative disease models [21,22,23]. Such computational analyses are widely employed to predict binding affinity, identify key ligand–protein interactions, and support the interpretation of experimental biological effects. These approaches provide valuable mechanistic insight into the potential molecular basis of drug activity and may facilitate the identification of novel therapeutic strategies.

Therefore, we hypothesized that the combination of metformin and caffeine may exert enhanced cytotoxic and pro-apoptotic effects compared with individual treatments through the modulation of metabolic and stress response pathways, including p53-associated signaling. The aim of this study was to evaluate the cytotoxic and antiproliferative effects of metformin and caffeine, individually and in combination, in human cancer cell lines and to investigate their potential interactions with the p53 protein using molecular docking analysis.

## 2. Results

The cytotoxic and antiproliferative effects of metformin and caffeine, administered individually and in combination, were evaluated in human cancer cell lines (*HeLa*, *A549*, and *HT29*), as well as in normal lung fibroblasts (*MRC-5*), using the sulforhodamine B (*SRB*) assay. Both compounds exhibited a dose- and time-dependent reduction in cell viability across all tested cancer cell lines. Among them, *HeLa* cells demonstrated the highest sensitivity to treatment, indicating a pronounced antiproliferative response.

Initial screening experiments revealed a significant inhibitory effect of metformin at concentrations ranging from 1 mM to 50 mM. Based on these findings, further analyses were conducted using a broader concentration range (50 μM to 10 mM) to better characterize the dose–response relationship. Comparable concentration ranges were applied to the *A549* and *HT29* cell lines.

Combined treatment exhibited variable effects depending on the cell line and exposure time, with limited synergistic interaction observed under specific conditions and predominantly antagonistic effects overall. Notably, cancer cell lines exhibited greater sensitivity to treatment than normal fibroblasts (*MRC-5*), indicating a degree of selectivity toward malignant cells.

Given the pronounced sensitivity of *HeLa* cells, subsequent mechanistic analyses focused on this cell line. Flow cytometry analysis confirmed the cytotoxic effects of metformin, while *ELISA*s demonstrated the modulation of p53 expression following treatment.

To further investigate the molecular basis of these effects, molecular docking studies were performed to evaluate the potential interactions of metformin and caffeine with the p53 protein. The docking results revealed that both compounds were capable of binding within the p53 structure, forming stabilizing interactions within the p53 binding site, which may provide mechanistic support for the experimental observations obtained in vitro.

### 2.1. Metformin

#### Cytotoxic Activity

The IC_50_ value determined after 24 h of metformin treatment was 6.036 mM in cervical carcinoma cells, 14.79 mM in lung adenocarcinoma cells, and 26.53 mM in colorectal adenocarcinoma cells. Since normal lung fibroblasts (*MRC-5*) were also tested under the same experimental conditions, the IC_50_ value after 24 h in these cells was 33.46 mM, which was considerably higher than those observed in tumor cell lines (Table 1).

**Figure 1:**
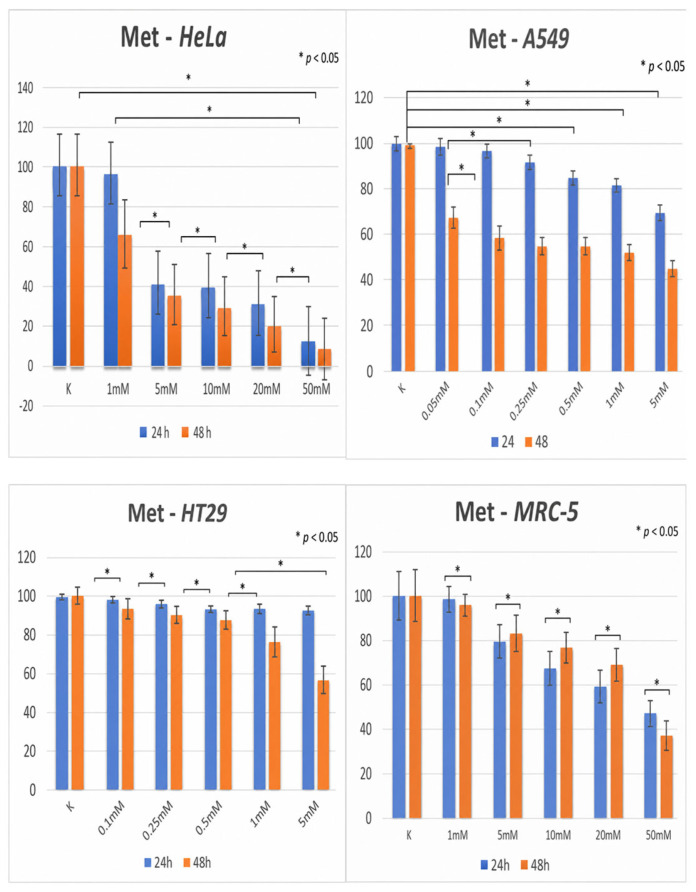
Effects of metformin on cell viability in *HeLa*, *A549*, *HT29*, and *MRC-5* cells following 24 h and 48 h treatment; data are presented as mean ± SD (*n* = 3), * *p* < 0.05.

As shown in Figure 1, *HeLa* cells exhibited the highest sensitivity to metformin treatment. After 48 h of exposure, all cell lines demonstrated a marked reduction in IC_50_ values, with an approximate 50% decrease compared with the values obtained after 24 h. Metformin reduced cell viability in a dose- and time-dependent manner across all tested cell lines. *HeLa* cells were the most sensitive, with an IC_50_ value of 2.28 mM at 48 h, followed by *A549* (3.30 mM) and *HT29* cells (10.54 mM). Although the concentrations of metformin used in this study were higher than those typically achieved in clinical settings, such concentrations are commonly employed in in vitro studies to elucidate underlying cellular and molecular mechanisms.

In contrast, normal *MRC-5* fibroblasts exhibited significantly higher IC_50_ values (33.46 mM), whereas cancer cell lines showed significantly lower IC_50_ values compared with *MRC-5* fibroblasts (*p* < 0.05), confirming selective cytotoxicity toward malignant cells.

### 2.2. Caffeine

**Figure 2:**
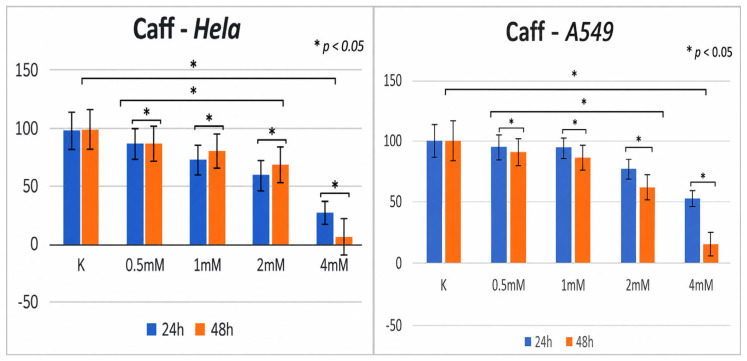

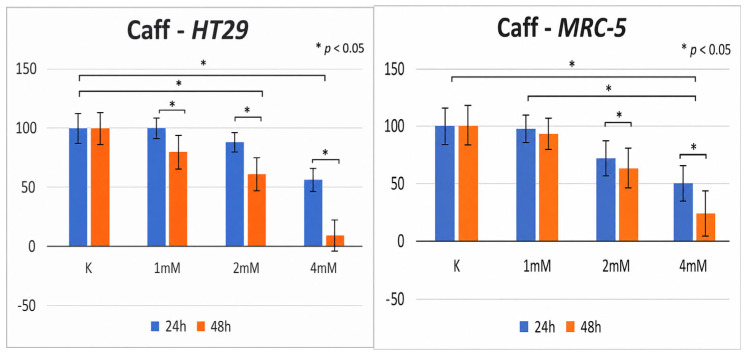
Cytotoxic activity of caffeine against all tested human cancer cell lines and *MRC-5*; data are presented as mean ± SD (*n* = 3), * *p* < 0.05.

Caffeine exhibited moderate antiproliferative activity in all tested cell lines. The strongest effects were observed in *HeLa* and *HT29* cells after 48 h, with IC_50_ values of 2.03 mM and 2.01 mM, respectively, as shown in Table 2, indicating a time-dependent enhancement in cytotoxic effects. Caffeine reduced cell viability in a dose- and time-dependent manner, although its effects were less pronounced than those of metformin in certain cell lines. Greater sensitivity to caffeine treatment was observed in *HeLa* and *HT29* cells (Figure 2).

Lower concentrations of caffeine did not produce significant effects and were therefore excluded from further analyses. Only concentrations ranging from 1 to 4 mM produced measurable effects and were consequently included in subsequent analyses.

### 2.3. Combination of Metformin and Caffeine

To investigate the cytotoxic effects of combined treatment with metformin and caffeine, concentrations were selected based on the results obtained from their individual applications. Cervical carcinoma (*HeLa*), lung adenocarcinoma (*A549*), and colorectal adenocarcinoma (*HT29*) cell lines were treated with metformin at concentrations ranging from 0.05 to 1 mM (0.05, 0.1, 0.25, 0.5, and 1 mM) in combination with caffeine (1 mM) to evaluate potential interactions affecting cell viability.

**Figure 3:**
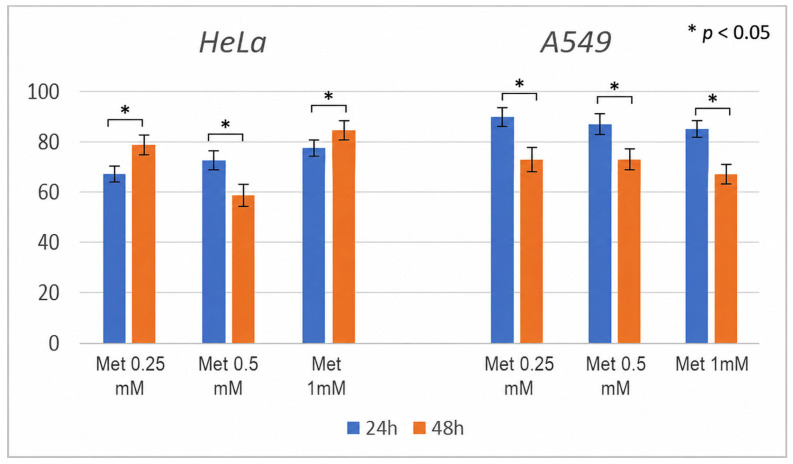
Cytotoxic effects of metformin and caffeine on *HeLa* and *A549* cell lines. Data are presented as mean ± SD (*n* = 3); * *p* < 0.05.

The results are presented in Table 3 and Figure 3. Combined treatment exhibited variable effects depending on the cell line and exposure time. The strongest cytotoxic effect was observed in *HeLa* cells, with *IC*_50_ values of 2.23 mM at 24 h and 2.40 mM at 48 h. In *A549* and *HT29* cells, higher *IC*_50_ values were observed, indicating lower sensitivity to the combined treatment. No consistent synergistic effects were observed across most experimental conditions, and the interaction between metformin and caffeine varied depending on the cell line and treatment duration.

An analysis of drug interactions using the combination index (*CI*) revealed that synergistic effects (*CI* < 1) were observed only in *HeLa* cells after 24 h, whereas antagonistic interactions predominated under most experimental conditions.

### 2.4. Mechanistic Studies

#### 2.4.1. Flow Cytometry

**Figure 4:**
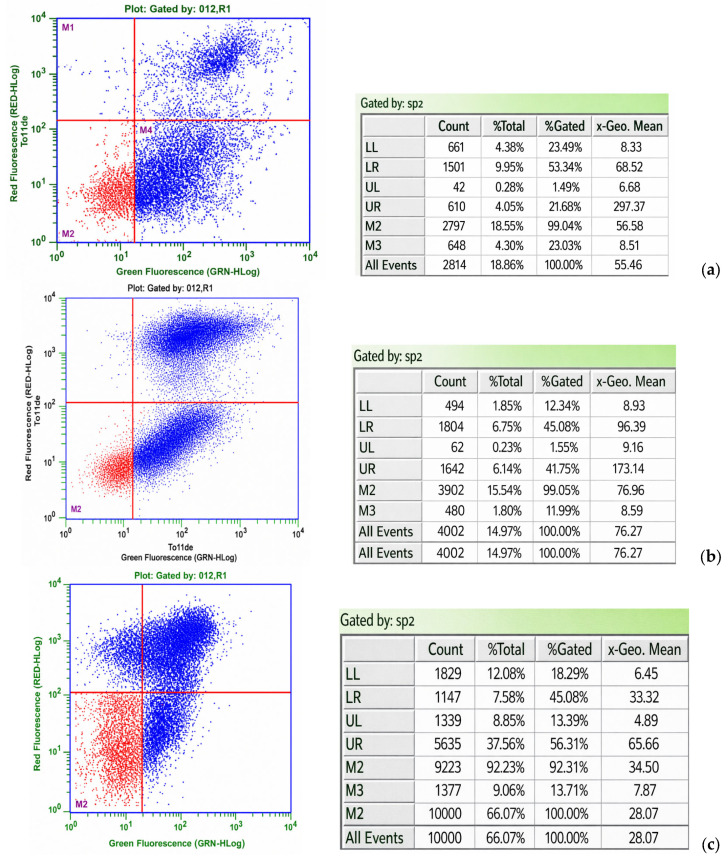
Apoptosis analysis in *HeLa* cells by flow cytometry following metformin treatment at 24 and 48 h. Representative dot plots illustrating viable, early apoptotic, and late apoptotic cell populations. Red-colored events indicate early apoptotic cells, whereas blue-colored events represent late apoptotic/secondary necrotic cells. (**a**) Control cells (without adjuvant) at 24 h; (**b**) metformin-treated cells at 24 h; and (**c**) metformin-treated cells at 48 h.

**Figure 5:**
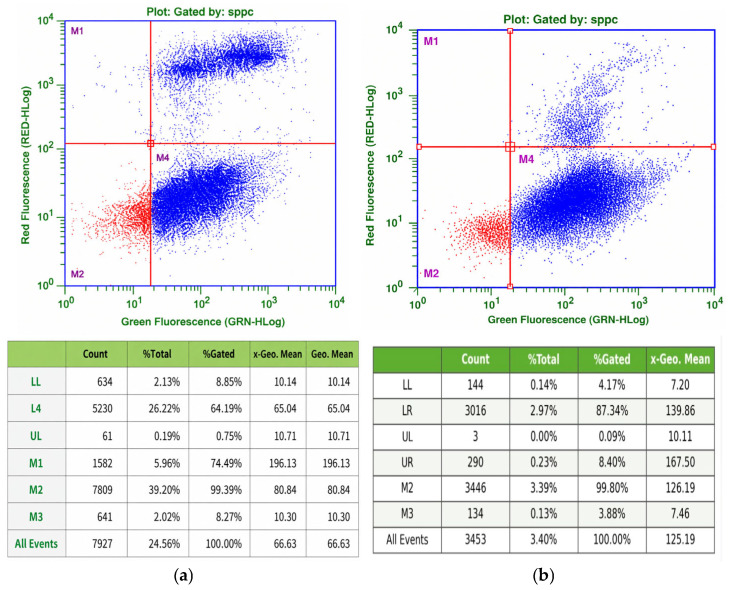
Flow cytometry dot plot analysis and population statistics in the *MRC-5* cell line following metformin treatment. Red-colored events indicate early apoptotic cells, whereas blue-colored events represent late apoptotic/secondary necrotic cells: (**a**) cells treated with metformin for 24 h; (**b**) cells treated with metformin for 48 h.

Apoptosis was assessed by flow cytometry, and representative histograms together with the corresponding quantitative data are presented in Figure 4. Flow cytometry analysis confirmed that metformin treatment increased the proportion of apoptotic cell populations in *HeLa* cells in a time-dependent manner. The proportions of both early and late apoptotic cells were higher after 48 h compared with 24 h, indicating the enhanced induction of apoptosis following prolonged exposure to metformin.In addition, apoptosis analysis in normal MRC-5 cells is presented in Figure 5, enabling comparison between tumor and non-tumor cell responses to metformin treatment.

#### 2.4.2. Immunofluorescence Microscopy Analysis of Apoptosis

**Figure 6:**
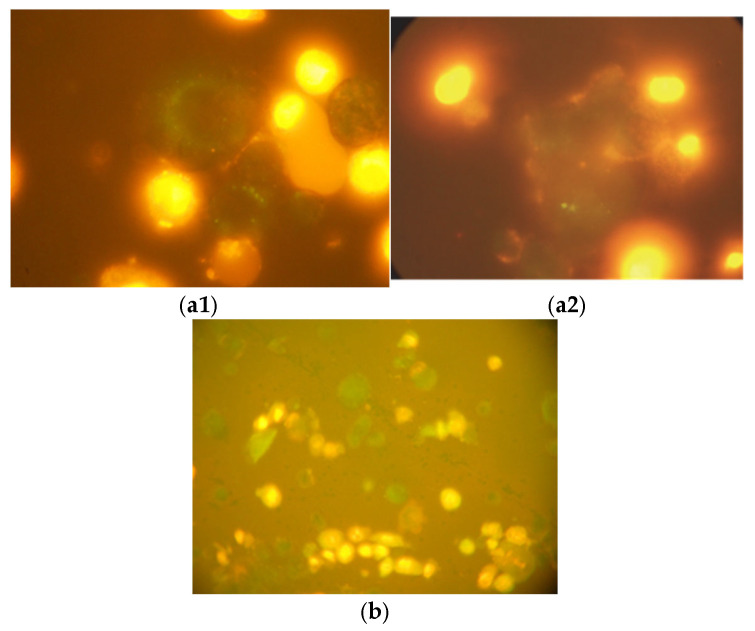
Apoptosis analysis in *HeLa* cells by immunofluorescence staining following metformin treatment: (**a1**,**a2**) immunofluorescence micrograph showing early apoptosis in cervical adenocarcinoma *HeLa* cells (1000× magnification); (**b**) immunofluorescence micrograph showing late apoptosis in cervical adenocarcinoma *HeLa* cells (400× magnification) ^1^.^1^ Footnote: Representative immunofluorescence image showing Annexin V-positive late apoptotic cells.

Immunofluorescence analysis using Annexin V staining confirmed the induction of apoptosis following metformin treatment. An increased proportion of apoptotic cells was observed, particularly after 48 h of exposure, supporting the results obtained by flow cytometry. In Figure 6(a1,a2), early apoptotic cells were identified based on positive Annexin V staining, indicating the initiation of programmed cell death. Differences in sensitivity among the tested cell lines were observed under the experimental conditions applied.

#### 2.4.3. p53 ELISA: Modulation of Tumor Suppressor p53 Expression Following Met and Combined Met–Caff Treatment in Cervical Cancer Cells (*HeLa* Cells)

**Figure 7:**
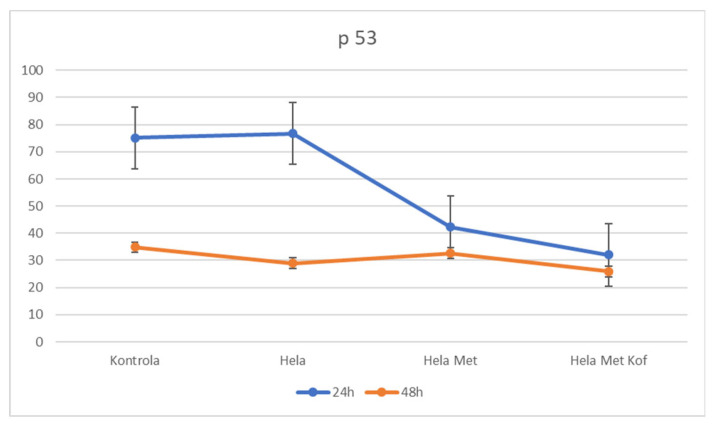
Quantitative measurement of p53 expression in HeLa cells after 24 h and 48 h of incubation.

Metformin treatment resulted in increased p53 expression in HeLa cells, which was further enhanced following combined treatment with caffeine (Figure 7).The observed increase in p53 expression following metformin treatment may be associated with the metabolic stress-induced activation of AMPK-related signaling pathways, ultimately contributing to apoptosis induction in *HeLa* cells. A complete dataset, including raw optical density values and calculated concentrations, is provided in the Supplementary Materials (Table S2).

The second and fourth columns represent the raw measured values, whereas the third and fifth columns correspond to concentrations calculated using the standard values and the calibration curve. O.D. denotes the optical density measured at 450 nm, and C represents the concentration expressed in international units per milliliter (U/mL). The calibration curve and the corresponding calculated concentrations are provided in the Supplementary Materials (Table S1 and Figure S1).

### 2.5. Molecular Docking Analysis

Rigid docking was initially performed to identify the key amino acid residues involved in ligand binding. The residues identified from the rigid docking poses were subsequently used to define flexible residues for the flexible docking protocol. Flexible docking yielded improved binding energies and more structurally consistent ligand conformations; therefore, final energetic and interaction-based interpretations were based on the flexible docking results.

In the DNA-bound p53–DNA model, caffeine exhibited the most favorable predicted non-covalent binding energy among the tested ligands, with a docking score of −6.15 kcal/mol and an estimated Ki value of 30.8 μM. Metformin showed a weaker but still favorable predicted interaction, with a docking score of −5.32 kcal/mol and an estimated Ki value of 125.3 μM.

To validate the docking protocol, the co-crystallized MQ ligand from the 6ZNC structure was re-docked into the experimentally defined ligand-binding region. The re-docked MQ pose reproduced the crystallographic binding orientation with an RMSD value of 2.07 Å relative to the experimental ligand position, which is within the commonly accepted threshold for successful docking pose reproduction. The re-docked MQ ligand exhibited a predicted binding energy of −5.06 kcal/mol and an estimated Ki value of 194.4 μM.

In the DNA-free p53 model, caffeine again exhibited a more favorable predicted binding energy than metformin. Caffeine was docked to DNA-free p53 with a binding energy of −7.91 kcal/mol and an estimated Ki value of 1.58 μM, whereas metformin showed a binding energy of −5.52 kcal/mol and an estimated Ki value of 89.4 μM (Table 4). Thus, caffeine retained the most favorable predicted non-covalent docking profile in both receptor contexts, while metformin exhibited moderate predicted binding in both the DNA-bound and DNA-free models.

The amino acid residues listed in Table 5 were selected from the initial rigid docking poses and subsequently used as flexible side chains in the AutoDock-GPU flexible docking protocol. Only local sidechain flexibility was considered, whereas the protein backbone and the remaining receptor residues were kept rigid. This approach enabled the limited local adaptation of the ligand-binding region while preserving the overall receptor conformation.

### 2.6. Drug Interaction Analysis by the Chou–Talalay Method

Drug interaction analysis performed at the IC_50_ level revealed that the effects of metformin combined with caffeine were both cell line- and time-dependent (Table 6). In *HeLa* cells, the combination showed a synergistic interaction after 24 h of treatment (CI = 0.779), whereas an antagonistic effect was observed after 48 h (CI = 1.545). In *A549* cells, antagonism was detected at both time points, with CI values of 1.134 at 24 h and 2.337 at 48 h. In *HT29* cells, the interaction was nearly additive after 24 h (CI = 0.998) but shifted toward antagonism after 48 h (CI = 1.775). In normal *MRC-5* fibroblasts, the combination showed slight synergy or a nearly additive effect after 24 h (CI = 0.957), whereas antagonism was observed after 48 h (CI = 1.526).

Overall, these findings indicate that the interaction between metformin and caffeine was not uniformly synergistic and depended strongly on both treatment duration and cellular background. Combination index (CI) values were calculated using the Chou–Talalay method implemented in CompuSyn software version 1.0. (ComboSyn Inc., Paramus, NJ, USA) based on IC_50_-derived dose equivalence.

#### Isobologram Analysis and Theoretical Model

**Figure 8:**
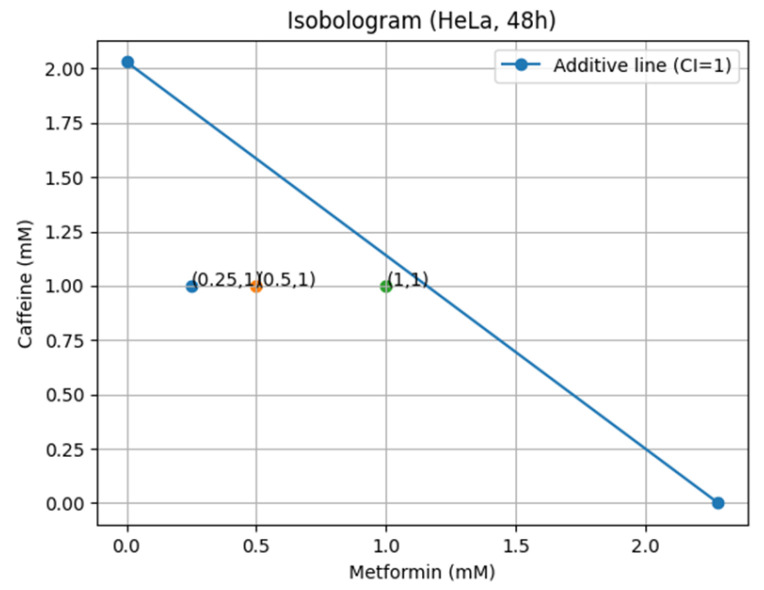
Isobologram analysis of interaction between metformin and caffeine in *HeLa* cells after 48 h of treatment.

The isobologram of metformin and caffeine interaction in *HeLa* cells after 48 h of treatment is presented in Figure 8. The straight line represents the theoretical additive effect (CI = 1), whereas data points located below the line indicate a synergistic interaction.

The CI values were consistent with the IC_50_ results, indicating that the effects of the combination varied depending on the cell line and treatment duration. Improved efficacy was observed only in *HeLa* cells after 24 h of treatment, whereas reduced efficacy predominated under the remaining experimental conditions.

## 3. Discussion

The present study investigated the cytotoxic and antiproliferative effects of metformin and caffeine, administered individually and in combination, in several human cancer cell lines, including cervical carcinoma (*HeLa*), lung adenocarcinoma (*A549*), and colorectal carcinoma (*HT29*). Our results demonstrated that metformin significantly reduced tumor cell viability in a dose- and time-dependent manner, whereas caffeine alone exhibited moderate antiproliferative activity. The combination of metformin and caffeine produced differential effects across the tested models, with synergism observed only under specific conditions, whereas antagonistic interactions predominated overall.

Metformin has attracted considerable attention as a potential candidate for drug repurposing in oncology because of its favorable safety profile and widespread clinical use in the treatment of type 2 diabetes mellitus. Epidemiological studies have suggested that diabetic patients receiving metformin therapy may exhibit a reduced risk of cancer development and improved survival outcomes compared with patients treated with other antidiabetic drugs [8,12]. These observations have stimulated extensive investigation into the anticancer mechanisms of metformin.

One of the primary mechanisms of metformin action involves the activation of AMP-activated protein kinase (*AMPK*), a key regulator of cellular energy homeostasis. The activation of AMPK results in the inhibition of the mammalian target of rapamycin (*mTOR*) signaling pathway, which plays a crucial role in cell growth, protein synthesis, and tumor cell proliferation [13,14,24]. The inhibition of *mTOR* signaling reduces tumor cell growth and promotes metabolic stress that may ultimately lead to apoptosis [18]. In addition to this mechanism, metformin has also been reported to inhibit mitochondrial respiratory chain complex I, resulting in decreased ATP production and the activation of metabolic stress responses in cancer cells [24,25]. The concentrations of metformin used in this study exceeded physiological plasma levels; however, such concentration ranges are commonly applied in in vitro models to overcome limited cellular uptake and to approximate the intracellular accumulation required to elicit measurable anticancer effects.

The predominance of antagonism represents a particularly important finding. One possible explanation is that the caffeine-mediated inhibition of checkpoint signaling may interfere with the cellular stress response required for the full activation of metformin-induced cytotoxicity. Specifically, while metformin is known to induce metabolic stress and activate AMPK–p53 signaling, caffeine may attenuate stress signaling pathways or alter cell cycle dynamics in a manner that reduces apoptotic susceptibility. Alternatively, caffeine-induced increases in intracellular cAMP levels may activate pro-survival pathways under certain conditions, thereby counteracting the effects of metabolic inhibition.

Another plausible explanation lies in the temporal dynamics of stress responses. The observed synergism at 24 h followed by antagonism at 48 h suggests that early co-treatment may transiently overwhelm cellular adaptive mechanisms, whereas prolonged exposure allows cancer cells to activate compensatory survival pathways. These findings highlight the importance of treatment scheduling and duration when evaluating drug combinations targeting cancer metabolism.

The antiproliferative effects observed in our study are consistent with previous findings demonstrating that metformin can inhibit the growth of multiple cancer types, including breast, pancreatic, lung, and colorectal cancers [26,27,28]. In the present study, metformin significantly reduced the viability of all investigated cancer cell lines. However, differences in sensitivity were observed among the cell lines. *HeLa* cells showed the highest responsiveness to metformin treatment, whereas *HT29* cells exhibited relatively greater resistance. Similar variability has been reported previously and may be related to differences in metabolic and signaling characteristics among tumor types [29].

Although the concentrations of metformin used in this study (up to 50 mM) exceeded physiological plasma levels (~10–40 µM), this approach is consistent with commonly applied in vitro experimental models. Higher extracellular concentrations are often required because of limited drug uptake under standard cell culture conditions, which lack the complex pharmacokinetic and tissue distribution mechanisms present in vivo.

Importantly, metformin is known to accumulate intracellularly, particularly within mitochondria, where it may reach concentrations substantially higher than those observed in plasma. This process is mediated, at least in part, by organic cation transporters (e.g., OCT1), which are variably expressed across different tumor types and may influence cellular sensitivity to metformin [30].

Nevertheless, the use of supraphysiological concentrations represents a limitation when considering clinical translation. Therefore, the present findings should be interpreted with caution, and further studies employing more physiologically relevant models, such as 3D cultures or in vivo systems, are warranted to better assess the therapeutic potential of metformin-based combinations.

In addition to metformin, caffeine has also been investigated for its potential role in modulating tumor cell survival. Caffeine is known to interfere with the DNA damage response by inhibiting checkpoint kinases such as ATM and ATR, which regulate cell cycle progression and DNA repair mechanisms [17]. The inhibition of these kinases disrupts the ability of tumor cells to repair DNA damage and increases susceptibility to apoptosis under conditions of cellular stress. Previous studies have demonstrated that caffeine can enhance the cytotoxic effects of anticancer therapies by sensitizing tumor cells to metabolic or genotoxic stress [16].

Although enhanced cytotoxicity was observed under limited conditions, the overall interaction was predominantly antagonistic. This observation suggests that caffeine may interfere with metformin-induced metabolic stress under certain conditions, potentially reducing its cytotoxic efficiency. Under specific conditions, the interaction between metformin and caffeine may result from complementary mechanisms. The simultaneous disruption of these pathways may overwhelm tumor cell survival mechanisms and promote apoptotic cell death.

An increased expression level of p53 was observed following treatment. The p53 protein is a key regulator of cell cycle arrest, DNA repair, and apoptosis in response to cellular stress [31]. The activation of p53 signaling pathways plays a crucial role in preventing malignant transformation and promoting programmed cell death. Previous studies have suggested that metabolic stress induced by metformin may stabilize p53 and enhance apoptotic signaling in cancer cells [32,33]. The increased expression of p53 observed in our experiments therefore supports the hypothesis that the activation of tumor suppressor pathways contributes to the cytotoxic effects of metformin and caffeine.

Recent evidence increasingly supports the involvement of p53-associated signaling pathways in the anticancer activity of metformin. Previous studies have demonstrated that metformin may regulate p53 through multiple mechanisms, including the activation of AMPK signaling, inhibition of mTOR pathways, modulation of p53 stability, and induction of apoptosis [32,34,35]. Previous studies have highlighted the close relationship between metformin-induced metabolic stress and p53-associated signaling pathways involved in tumor suppression and apoptosis [33]. Yi et al. reported that metformin induces apoptosis through the regulation of p53 and its downstream target genes, including DEC1, further supporting the role of p53-mediated stress responses in metformin-induced cytotoxicity [35]. In addition, comprehensive reviews have highlighted the close relationship between metformin and the p53 protein family (p53/p63/p73), emphasizing that AMPK activation may contribute to tumor suppression through the downstream modulation of p53-dependent pathways [31,34]. Interestingly, recent findings by Wu et al. demonstrated that metformin may also promote the degradation of the mutant p53 protein in pancreatic cancer cells via SIRT1-mediated deacetylation mechanisms, leading to the inhibition of tumor growth and increased apoptosis [36]. Similarly, studies in breast cancer models showed that metformin-induced growth inhibition and apoptosis were significantly associated with p53 activation and AMPK–mTOR signaling pathways [37].

These findings are consistent with the increased p53 expression observed in the present study and further support the hypothesis that p53-related stress signaling contributes to the antiproliferative effects of metformin and metformin–caffeine treatment. Considering that HeLa cells are characterized by the HPV E6-mediated functional suppression of p53, the observed increase in p53 expression following treatment may indicate the partial restoration of stress response signaling pathways under conditions of metabolic stress [38].

However, the precise role of p53 modulation may depend on tumor type, the mutational status of p53, and treatment conditions, indicating that further mechanistic studies are required to fully clarify the interaction between metformin, caffeine, and p53-associated pathways [38]. This apparent dual effect may be explained by differences in p53 mutational status, since metformin has been reported to stabilize wild-type p53 while promoting the degradation of oncogenic mutant p53 proteins in certain cancer models [36].

An additional important observation of this study is the relatively low cytotoxic effect observed in normal lung fibroblasts (*MRC-5*). While metformin and caffeine significantly inhibited the proliferation of cancer cell lines, their effects on normal cells were considerably less pronounced. This finding suggests a degree of selectivity toward malignant cells. Tumor cells often exhibit altered metabolic pathways and increased energy demands compared with normal cells, making them more susceptible to metabolic inhibitors such as metformin [27]. Importantly, the observed shift from synergism at 24 h to antagonism at 48 h highlights the critical role of treatment timing in determining the outcome of metabolic drug combinations.

Recent studies have further highlighted the potential of metformin as a metabolic modulator in cancer therapy. For example, Heckman-Stoddard et al. reported that metformin can influence tumor metabolism and inhibit cancer cell growth through both systemic metabolic effects and direct cellular mechanisms, supporting its role as a promising candidate for drug repurposing in oncology [26]. Similarly, Rena et al. provided an updated overview of the molecular mechanisms underlying metformin action and emphasized its ability to interfere with mitochondrial respiration and cellular energy balance, which may selectively affect tumor cells with high metabolic demands [29].

More recently, clinical and translational studies have continued to explore the anticancer potential of metformin in various malignancies. A comprehensive review by Marciniak et al. highlighted the growing evidence supporting the use of metformin as an adjuvant therapy in cancer treatment, particularly in tumors characterized by altered metabolic signaling pathways [34]. In addition, emerging studies suggest that metabolic modulators such as metformin may enhance the sensitivity of cancer cells to other stress-inducing agents, thereby improving therapeutic outcomes [22].

These findings support the hypothesis proposed in the present study that metabolic stress induced by metformin may increase tumor cell susceptibility to additional modulators such as caffeine. The combination of agents targeting different cellular pathways may result in complex and sometimes antagonistic interactions, underscoring the need for a careful evaluation of combination strategies in cancer therapy.

Despite these promising findings, several limitations should be acknowledged. The experiments were conducted under in vitro conditions and therefore do not fully reproduce the complexity of tumor biology in vivo. In addition, although increased p53 expression suggests the activation of tumor suppressor pathways, further molecular studies are required to clarify the precise signaling mechanisms responsible for the observed interaction between metformin and caffeine.

The molecular docking results provide additional insight into the potential interactions between metformin, caffeine, and the p53 protein. Interestingly, caffeine exhibited a higher binding affinity (−5.2 kcal/mol) than metformin (−4.1 kcal/mol), which can be attributed to its aromatic structure and capacity to form π–π and hydrophobic interactions within the protein binding site.

However, despite this stronger predicted binding, caffeine demonstrated weaker antiproliferative effects in vitro compared with metformin. This apparent discrepancy suggests that binding affinity alone is not the primary determinant of biological activity in this system.

Metformin is known to exert its anticancer effects predominantly through indirect mechanisms, including the induction of metabolic stress, activation of AMPK, and modulation of downstream signaling pathways such as mTOR and p53. Therefore, its relatively low docking affinity is consistent with a mechanism that does not rely on strong direct binding to target proteins.

In contrast, although caffeine is capable of interacting more favorably with the p53 structure at the molecular level, its biological effects appear to depend on the modulation of signaling pathways such as ATM/ATR and cAMP-mediated processes, which may not directly translate into strong antiproliferative activity under all experimental conditions.

The successful re-docking of the co-crystallized MQ ligand, with an RMSD of 2.07 Å relative to the crystallographic pose, supports the reliability of the applied docking protocol for reproducing the experimentally observed ligand-binding orientation in the p53-DNA model. This is particularly important because the same grid definition and docking settings were subsequently applied to caffeine and metformin, allowing for a consistent comparison of their predicted binding behavior. Although docking scores should not be interpreted as direct experimental binding affinities, the validated re-docking result increases confidence that the predicted poses of caffeine and metformin reflect plausible ligand accommodation within the selected p53 binding regions.

Therefore, the more favorable binding energy calculated for caffeine, together with its stabilizing hydrogen bonding, hydrophobic, and van der Waals interactions, suggests a greater potential for non-covalent interaction with p53-containing models than metformin.

**Figure 9:**
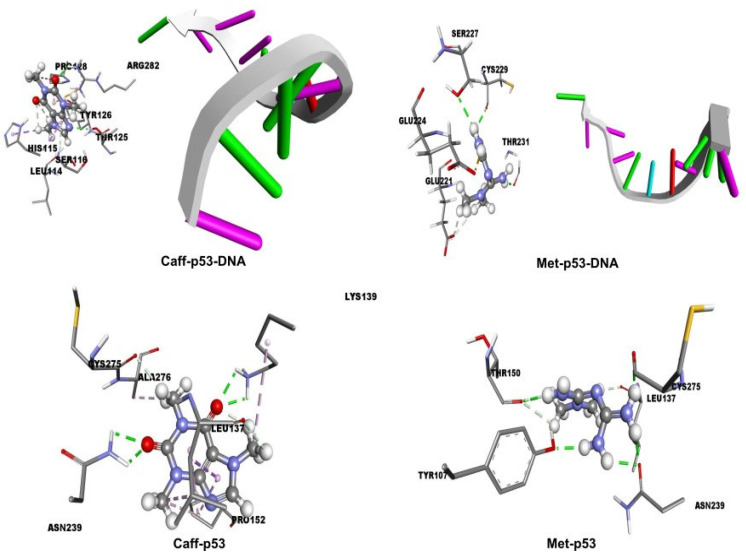
Flexible docking poses of caffeine and metformin in p53-DNA and ligand-free p53 receptor models.

The interaction profile was consistent with the structural properties of the two compounds. Caffeine, which contains an aromatic xanthine scaffold, formed stabilizing contacts within the p53 binding region, including hydrogen bonding and non-polar contacts with residues located near the binding pocket. In the p53-DNA model, caffeine was positioned near residues including His115, Ser116, Leu114, Tyr126, Thr125, Pro128, and Arg282, suggesting a binding mode supported by hydrogen bonding, hydrophobic contacts, van der Waals interactions, and possible aromatic stabilization. In the DNA-free p53 model, caffeine was positioned near residues including Asn239, Cys275, Ala276, Leu137, and Pro152, supporting a binding mode stabilized by hydrogen bonding, hydrophobic contacts, and van der Waals interactions (Figure 9).

In contrast, Met interacted predominantly through polar contacts and hydrogen bonds, consistent with its hydrophilic character. In the p53-DNA model, metformin formed polar interactions with residues including Glu224, Cys229, Ser227, and Thr231, whereas in the DNA-free p53 model, it interacted with residues including Thr150, Tyr107, Leu137, Cys275, and Asn239.

Overall, the flexible docking results suggest that both caffeine and metformin can form plausible non-covalent interactions with p53-containing receptor models. Caffeine exhibited more favorable binding affinity than metformin in both the DNA-bound and DNA-free models, whereas metformin displayed a more polar interaction profile dominated by hydrogen bonding. These findings provide structural support for possible p53-interacting or p53-modulating behavior; however, they do not, by themselves, demonstrate direct biological activity or direct inhibition of p53.

Overall, the results of this study support the concept of drug repurposing in oncology [39], highlighting metformin as a primary contributor to antiproliferative effects. The interaction between metformin and caffeine was not uniformly beneficial and depended on treatment duration and cellular context. These findings underscore the complexity of metabolic drug interactions and emphasize the need for further studies using physiologically relevant models to evaluate their therapeutic potential.

## 4. Materials and Methods

### 4.1. Reagents

Metformin hydrochloride (99.99%, Galenika, Belgrade, Serbia) was dissolved in phosphate-buffered saline (PBS; Dulbecco’s PBS, Capricorn Scientific GmbH, Ebsdorfergrund, Germany) immediately before use. The following reagents were used: 2-deoxy-D-glucose (2DG) and caffeine (Abcam, Cambridge, UK); sulforhodamine B, Tris base, dimethyl sulfoxide (DMSO), and DMEM (Sigma-Aldrich Chemie GmbH, Taufkirchen, Germany); trichloroacetic acid (Merck Chemie GmbH, Darmstadt, Germany); acetic acid and sodium chloride (Zorka Pharma Hemija, Šabac, Serbia); Annexin V-FITC and propidium iodide (Becton Dickinson Pharmingen, Heidelberg, Germany); a viability kit (Invitrogen, Carlsbad, CA, USA); and SYBR-14/PI (Sigma-Aldrich Chemie GmbH, Taufkirchen, Germany). Metformin and other pharmacological modulators were freshly prepared and added immediately prior to treatment.

### 4.2. Cell Culture

Human cervical carcinoma *HeLa* cells (ATCC^®^ CCL-2™), *A549* lung adenocarcinoma cells (ATCC^®^ CCL-185™), and *HT29* colorectal carcinoma cells (ATCC^®^ HTB-38™) were obtained from the American Type Culture Collection (ATCC, Manassas, VA, USA). Cells were cultured in Dulbecco’s Modified Eagle Medium (DMEM) containing 4.5 g/L glucose and supplemented with 10% fetal bovine serum (FBS) and 1% antibiotic–antimycotic solution.

Cells were maintained at 37 °C in a humidified atmosphere containing 5% CO_2_. Cell lines were passaged twice weekly and used during the exponential growth phase (passages 3–10). After thawing, cells were cultured in 25 cm^2^ flasks at a density of 1 × 10^6^ cells in 10 mL of medium. Upon reaching confluence, cells were detached using 0.1% trypsin and seeded for subsequent experiments.

For viability assays, cells were seeded in 96-well plates at a density of 1 × 10^5^ cells/well, whereas for flow cytometry analysis, cells were seeded in 25 cm^2^ flasks at a density of 1 × 10^6^ cells/flask.

### 4.3. Cell Viability and Proliferation Assays

#### 4.3.1. Trypan Blue Exclusion Assay

Only viable cells were used in the experiments. Cell viability was determined using the Trypan Blue exclusion assay [40]. Briefly, cells were mixed with 0.1% Trypan Blue and counted using a hemocytometer under an inverted microscope. Cell viability was assessed prior to treatment to ensure that only viable cells were included in subsequent experiments.

#### 4.3.2. Sulforhodamine B (SRB) Assay

The cytotoxic effects of metformin and caffeine on the proliferation of HeLa, A549, and HT29 cells were evaluated using the sulforhodamine B (SRB) assay. Cells in the exponential growth phase were treated with increasing concentrations of metformin (0.05–50 mM) and caffeine (0.5–5 mM), either individually or in combination, for 24 and 48 h. The SRB assay is based on the quantification of cellular protein content through the binding of sulforhodamine B to amino acid residues [41]. Following treatment, cells were fixed with 10% trichloroacetic acid, stained with SRB, and washed with 1% acetic acid. The bound dye was then solubilized, and absorbance was measured at 540 nm [42].

### 4.4. Apoptosis Analysis by Flow Cytometry

Apoptosis was assessed using Annexin V-FITC/propidium iodide (PI) staining. Following treatment, cells were trypsinized, collected, and centrifuged at 250× *g* for 5 min. The cell pellet was then resuspended in binding buffer and stained with Annexin V-FITC and PI. Samples were incubated for 15 min in the dark at room temperature and analyzed within 1 h using a Guava EasyCyte flow cytometer (Guava Technologies, Hayward, CA, USA). Annexin V-positive/PI-negative cells were classified as early apoptotic, whereas Annexin V-positive/PI-positive cells were considered late apoptotic or necrotic [43]. Staining was performed according to the manufacturer’s instructions (BD Pharmingen, San Diego, CA, USA).

Cell death parameters were analyzed by Annexin V-FITC/PI flow cytometry FlowJo software version 10.0 (FlowJo LLC, Ashland, OR, USA). At least 10,000 events were acquired per sample. Representative cell cultures were photographed using an Olympus BX40 microscope equipped with an Olympus SP-500 UZ camera, and images were ar-chived using Olympus Master 1.31 software (Olympus, Tokyo, Japan).

### 4.5. ELISA: Determination of Tumor Suppressor Genes for p53

The expression of the tumor suppressor protein p53 in HeLa cells was quantified using a commercially available ELISA kit (Abcam, Cambridge, UK) according to the manufacturer’s instructions. Briefly, standards, control samples, and treated cell lysates were added to 96-well microplates and incubated with a biotinylated anti-p53 antibody. Following the washing steps, streptavidin–horseradish peroxidase (HRP) conjugate was added and incubated under standard conditions. After the removal of unbound components, tetramethylbenzidine (TMB) substrate was applied, producing a colorimetric reaction proportional to the p53 concentration. The reaction was terminated with a stop solution, and absorbance was measured at 450 nm using a microplate reader. p53 concentrations were determined from a standard calibration curve generated using known concentrations of the protein.

All measurements were performed in triplicate, and the results are presented as the mean ± standard deviation (SD).

### 4.6. Molecular Docking Analysis

#### Receptor and Ligand Preparation

Two complementary p53 receptor models were employed for molecular docking studies. Receptor structures were obtained in PDB format from the RCSB Protein Data Bank (accessed on 2 May 2026): the DNA-bound p53–DNA complex 6ZNC, containing the co-crystallized methylene quinuclidinone (MQ) ligand [44], and the DNA-free, ligand-free p53 core-domain structure 2OCJ [45]. The 6ZNC structure was used to evaluate ligand binding in the context of DNA-bound p53 and to define a structurally characterized ligand-binding region, whereas 2OCJ served as a complementary DNA-free model for assessing ligand interactions in the absence of both DNA and a native small-molecule ligand.

Receptor preparation involved the removal of non-essential heteroatoms, water molecules, and co-crystallized ligands using BIOVIA Discovery Studio 4.0 [46]. Polar hydrogen atoms were added, and Kollman partial charges were assigned using AutoDockTools (ADT) version 1.5.6 and performed using AutoDock4.2 [47]. The ligands caffeine (Caff), metformin (Met), and MQ were prepared and converted into PDBQT format using Open Babel [48].

Docking grids were centered on the ligand-binding regions identified for each receptor model. Grid center coordinates (x, y, z; Å) were set to 167.573, −7.263, 33.461 for the p53–DNA model based on 6ZNC and 3.817, 3.325, 57.304 for the DNA-free p53 model based on 2OCJ. Docking simulations were carried out using AutoDock-GPU [49]. For each ligand, 200 genetic algorithm runs were performed (--nrun 200), with 2,500,000 energy evaluations (--nev 2,500,000), 42,000 generations (--ngen 42,000), a population size of 150, and ADADELTA local search parameters.

Both rigid and flexible docking protocols were applied. Initial rigid docking was conducted to identify the preferred binding regions and amino acid residues involved in ligand recognition. These residues were subsequently defined as flexible residues in the flexible docking protocol. Because flexible docking yielded more favorable binding energies and more plausible interaction geometries, the final docking results and interpretation were based on the flexible docking analysis [50,51]. Caffeine, metformin, and MQ were docked independently; simultaneous dual-ligand docking was not performed.

Protein–ligand interactions were analyzed using BIOVIA Discovery Studio and visual inspection, including hydrogen bonding, hydrophobic interactions, van der Waals contacts, and aromatic interactions. The docking protocol was validated by re-docking the co-crystallized MQ ligand into the 6ZNC binding site, followed by the calculation of the root mean square deviation (RMSD) between the experimental and re-docked ligand conformations.

### 4.7. Statistics

All experiments were performed in at least three independent replicates, and the results are presented as the mean ± standard deviation (SD). Statistical analysis was conducted using a one-way analysis of variance (ANOVA) followed by Tukey’s post hoc test for multiple comparisons. Comparisons were made between treated groups and the control group, as well as between combination treatments and their respective individual treatments. Differences were considered statistically significant at *p* < 0.05.

IC50 values were determined by nonlinear regression analysis using a log(inhibitor) versus response model. The interaction between metformin and caffeine was evaluated using the Chou–Talalay method [52,53]. Combination index (CI) values were calculated using CalcuSyn software version 2.1 (Biosoft, Cambridge, UK) based on the median-effect principle to determine synergistic, additive, or antagonistic effects.

## 5. Conclusions

This study demonstrated that caffeine does not uniformly enhance the efficacy of metformin but may instead induce antagonistic interactions depending on treatment duration and cellular context. The combination treatment produced variable effects across different cell types and exposure times, with limited synergistic interactions observed only under specific conditions, while predominantly antagonistic effects were detected overall. The induction of apoptosis and increased p53 expression suggest the involvement of tumor-suppressive pathways, whereas the limited toxicity observed in normal fibroblasts indicates a degree of selectivity toward malignant cells. These findings support the potential repurposing of metformin as an anticancer agent; however, the addition of caffeine does not consistently improve its anticancer activity and may result in antagonistic interactions under certain experimental conditions. Further in vivo studies and mechanistic investigations are required to confirm the clinical relevance of these findings and to elucidate the underlying molecular mechanisms.

Flexible molecular docking analysis demonstrated that both caffeine and metformin are capable of forming plausible non-covalent interactions with p53 receptor models, with caffeine consistently exhibiting more favorable binding energies in both DNA-bound and DNA-free conformations. The successful re-docking of the co-crystallized MQ ligand confirmed the reliability and validity of the applied docking protocol. These findings suggest a greater potential of caffeine for p53-related interactions; however, additional experimental studies are necessary to verify their biological significance.

## Figures and Tables

**Table 1:** The IC_50_ value (mM) following 24 and 48 h of metformin treatment in *HeLa* (cervical carcinoma), *A549* (lung adenocarcinoma), and *HT29* (colorectal carcinoma).

Cell Line	Metformin 24 h	Metformin 48 h
*HeLa*	6.04	2.28
*A549*	14.79	3.30
*HT29*	26.53	10.54
*MRC-5*	33.46 ^1^	33.46 ^1^

Abbreviations: ^1^ normal lung fibroblasts (*MRC-5*)—control cell line.

**Table 2:** The IC_50_ value mM determined after 24 and 48 h of caffeine treatment in *HeLa* (cervical carcinoma), *A549* (lung adenocarcinoma), and *HT29* (colorectal carcinoma).

Cell Line	Caffeine 24 h	Caffeine 48 h
HeLa	2.44	2.03
A549	3.38	2.44
HT29	3.41	2.01
MRC-5	3.55	2.62

Abbreviations: normal lung fibroblasts (MRC-5)—control cell line.

**Table 3:** The IC_50_ value determined after 24 and 48 h of (Met + Caff) treatment in *HeLa* (cervical carcinoma), *A549* (lung adenocarcinoma), and *HT29* (colorectal carcinoma).

Cell Line	24 h	48 h
*HeLa*	2.23	2.40
*A549*	12.39	6.36
*HT29*	18.71	13.46
*MRC-5*	22.60	38.30

Abbreviations: Met + Caff, metformin and caffeine; normal lung fibroblasts (*MRC-5*)—control cell line.

**Table 4:** Flexible docking binding energies (ΔG) and estimated inhibition constants (Ki) for MQ, caffeine, and metformin in p53-DNA and DNA-free p53 receptor models.

p53 Receptor Models	ΔG (kcal/mol)	Ki (μM)
MQ-p53-DNA	−5.06	194.4
Caff-p53-DNA	−6.15	30.8
Met-p53-DNA	−5.32	125.3
Caff-p53	−7.91	1.6
Met-p53	−5.52	89.4

Footnote: MQ—methylene quinuclidinone (MQ) ligand; Caff—caffeine; Met—metformin.

**Table 5:** The flexible residues selected for the AutoDock-GPU flexible docking protocol based on the initial rigid docking poses.

Receptor Model	PDB ID *	Ligand	Flexible Residues Selected for Docking
p53-DNA model	6ZNC **	Caff	His115, Ser116, Leu114, Tyr126, Thr125, Pro128, Arg282
p53-DNA model	6ZNC	Met	Glu224, Ser227, Cys229, Thr231
DNA-free p53 model	2OCJ ***	Caff	Asn239, Cys275, Ala276, Leu137, Pro152
DNA-free p53 model	2OCJ	Met	Thr150, Tyr107, Leu137, Cys275, Asn239

Footnote: PDB ID *—Protein Date Bank Identification Code; 6ZNC **—experimentally determined p-53 structure receptor in DNA-bound p53 model; 2OCJ ***—experimentally determined p-53 structure in DNA-free p53 model; Caff—caffeine; Met—metformin.

**Table 6:** Evaluation of drug interactions between metformin and caffeine based on IC_50_-derived combination index (CI) values in *HeLa*, A549, HT29, and MRC-5 cells after 24 h and 48 h of treatment.

	24 h	48 h
	CI ^1^	CI ^1^
*HeLa*	0.78	1.55
*A549*	1.13	2.34
*HT29*	0.99	1.78
*MRC-5*	0.96	1.53

^1^ Footnote: CI < 1 indicates synergism, CI = 1 additive effect, and CI > 1 antagonism.

## Data Availability

The datasets generated during this study are not publicly available due to privacy restrictions but are available from the corresponding author on reasonable request.

## Supplementary Materials

The following supporting information can be downloaded at https://www.mdpi.com/article/10.3390/molecules31111877/s1, Figure S1: Calibration curve for p53 determination (https://www.abcam.com); Figure S2: Molecular docking of metformin and caffeine to p53. Table S1: Standards for calibration curve for p53 (https://www.abcam.com). Table S2: Comparative Measurements in HeLa and MRC-5 Cells Following 24 h and 48 h of Incubation.

## References

[1] World Health Organization (2024). World Health Statistics 2024: Monitoring Health for the SDGs.

[2] Wagle N.S., Nogueira L., Devasia T.P., Mariotto A.B., Yabroff K.R., Islami F., Jemal A., Alteri R., Ganz P.A., Siegel R.L. (2025). Cancer treatment and survivorship statistics 2025. CA Cancer J. Clin..

[3] Siegel R.L., Kratzer T.B., Giaquinto A.N., Sung H., Jemal A. (2025). Cancer statistics 2025. CA Cancer J. Clin..

[4] Bray F., Laversanne M., Sung H., Ferlay J., Siegel R.L., Soerjomataram I., Jemal A. (2024). Global cancer statistics 2022: GLOBOCAN estimates of incidence and mortality worldwide for 36 cancers in 185 countries. CA Cancer J. Clin..

[5] Pryor R., Cabreiro F. (2015). Repurposing metformin: An old drug with new tricks in its binding pockets. Biochem. J..

[6] Flory J., Lipska K. (2019). Metformin in 2019. JAMA.

[7] Evans J.M.M., Donnelly L.A., Emslie-Smith A.M., Alessi D.R., Morris A.D. (2005). Metformin and reduced risk of cancer in diabetic patients. BMJ.

[8] Quinn B.J., Kitagawa H., Memmott R.M., Gills J.J., Dennis P.A. (2013). Repositioning metformin for cancer prevention and treatment. Trends Endocrinol. Metab..

[9] Vujovic S., Perovic S., Vlaovic M., Scepanovic A., Scepanovic S. (2026). From metabolism to longevity: Molecular mechanisms underlying metformin’s anticancer and anti-aging effects. Curr. Issues Mol. Biol..

[10] Saraei P., Asadi I., Kakar M.A., Moradi-Kor N. (2019). The beneficial effects of metformin on cancer prevention and therapy: A comprehensive review of recent advances. Cancer Manag. Res..

[11] Boroughs L.K., DeBerardinis R.J. (2015). Metabolic pathways promoting cancer cell survival and growth. Nat. Cell Biol..

[12] Morales D.R., Morris A.D. (2015). Metformin in cancer treatment and prevention. Annu. Rev. Med..

[13] Zakikhani M., Dowling R., Fantus I., Sonenberg N., Pollak M. (2006). Metformin is an AMP kinase-dependent growth inhibitor for breast cancer cells. Cancer Res..

[14] Dowling R.J.O., Zakikhani M., Fantus I.G., Pollak M., Sonenberg N. (2007). Metformin inhibits mTOR signaling in cancer cells. Cancer Res..

[15] Wang Z., Gu C., Wang X., Lang Y., Wu Y., Wu X., Zhu X., Wang K., Yang H. (2019). Caffeine enhances the anti-tumor effect of 5-fluorouracil via increasing the production of reactive oxygen species in hepatocellular carcinoma. Med. Oncol..

[16] Bode A.M., Dong Z. (2007). The enigmatic effects of caffeine in cell cycle and cancer. Cancer Lett..

[17] Weber A.M., Ryan A.J. (2015). ATM and ATR as therapeutic targets in cancer. Pharmacol. Ther..

[18] Fagundes T.R., Madeira T.B., Melo G.P., Bordini H.P., Marinello P.C., Nixdorf S.L., Cecchini A.L., Luiz R.C. (2022). Caffeine improves the cytotoxic effect of dacarbazine on B16F10 murine melanoma cells. Bioorg. Chem..

[19] Al-Lazikani B., Banerji U., Workman P. (2012). Combinatorial drug therapy for cancer in the post-genomic era. Nat. Biotechnol..

[20] Trott O., Olson A.J. (2010). AutoDock Vina: Improving the speed and accuracy of docking with a new scoring function, efficient optimization, and multithreading. J. Comput. Chem..

[21] Chandra P. (2025). Molecular docking insights and antioxidant activity of isolated bioactive compounds from *Stevia rebaudiana* leaves. Prospect. Pharm. Sci..

[22] Surana K., Jadhav S., Khairnar R., Ahire E., Kasar G. (2025). In silico prediction of some indole derivatives against VirB8 from *Brucella suis* and cyanobacterial membrane-bound manganese superoxide dismutase. Prospect. Pharm. Sci..

[23] Culletta G., Zappalà M., Ettari R., Almerico A.M., Tutone M. (2021). Immunoproteasome and non-covalent inhibition: Exploration by advanced molecular dynamics and docking methods. Molecules.

[24] Hardie D.G. (2012). AMPK: A key regulator of energy balance in cells and organisms. Nat. Rev. Mol. Cell Biol..

[25] Wheaton W.W., Weinberg S.E., Hamanaka R.B., Soberanes S., Sullivan L.B., Anso E., Glasauer A., Dufour E., Mutlu G.M., Budigner G.S. (2014). Metformin inhibits mitochondrial complex I of cancer cellsto reduce tumorigenesis. eLife.

[26] Heckman-Stoddard B., DeCensi A., Sahasrabuddhe V., Ford L. (2017). Repurposing metformin for cancer prevention and treatment. Diabetologia.

[27] Pernicova I., Korbonits M. (2014). Metformin—Mode of action and implications for cancer therapy. Endocr. Rev..

[28] Kasznicki J., Sliwinska A., Drzewoski J. (2014). Metformin in cancer prevention and therapy. Ann. Transl. Med..

[29] Rena G., Hardie D.G., Pearson E.R. (2017). The mechanisms of action of metformin. Diabetologia.

[30] Luengo A., Sullivan L.B., Vander Heiden M.G. (2014). Understanding the complexity of metformin action: Limiting mitochondrial respiration to improve cancer therapy. BMC Biol..

[31] Vousden K.H., Lane D.P. (2007). p53 in health and disease. Nat. Rev. Mol. Cell Biol..

[32] Wang Y., Xu W., Yan Z., Zhao W., Mi J., Li J., Yan H. (2018). Metformin induces autophagy and G0/G1 cell cycle arrest via regulating the AMPK/mTOR/p70S6K and p53 signaling pathways in human pancreatic cancer cells. J. Exp. Clin. Cancer Res..

[33] Buzzai M., Jones R.G., Amaravadi R.K., Lum J.J., DeBerardinis R.J., Zhao F., Viollet B., Thompson C.B. (2007). Systemic treatment with the antidiabetic drug metformin selectively impairs p53-deficient tumor cell growth. Cancer Res..

[34] Marciniak M., Torbicki A., Korpalski M., Pawluczyk M., Pawlikowski K., Żygłowicz M., Augustyn D., Gaworek P., Trybuła A. (2025). Metformin in oncology—Its effect on cancer development and progression. Med. Sci..

[35] Yi G., He Z., Zhou X., Xian L., Yuan T., Jia X., Hong J., He L., Liu J., Fan D. (2015). Metformin induces apoptosis through the downregulation of p53-dependent differentiated embryo chondrocyte 1 in human cervical carcinoma cells. Biochem. Biophys. Res. Commun..

[36] Wu G., Zhang M., Meng Y., Ying Y., Zhang S., Chen M., Li D., Yang S., Luo M. (2023). Metformin inhibits the growth of pancreatic cancer cells by inducing degradation of mutant p53 protein. Res. Sq..

[37] Zhuang Y., Miskimins W.K. (2011). Metformin induces both caspase-dependent and poly(ADP-ribose) polymerase-dependent cell death in breast cancer cells. Mol. Cancer Res..

[38] Kalender A., Selvaraj A., Kim S.Y., Gulati P., Brûlé S., Viollet B., Kemp B.E., Bardeesy N., Dennis P., Schlager J.J. (2010). Metformin, independent of AMPK, inhibits mTORC1 in a Rag GTPase-dependent manner. Cell Metab..

[39] Lefranc F., Tabanca N., Kiss R. (2017). Assessing the anticancer effects associated with food products and/or nutraceuticals using in vitro and in vivo preclinical development-related pharmacological tests. Semin. Cancer Biol..

[40] Strober W. (2001). Trypan blue exclusion test of cell viability. Curr. Protoc. Immunol..

[41] Skehan P., Storeng R., Scudiero D., Monks A., McMahon J., Vistica D., Warren J.T., Bokesch H., Kenney S., Boyd M.R. (1990). New colorimetric cytotoxicity assay for anticancer-drug screening. J. Natl. Cancer Inst..

[42] Bogdanović G., Raletić-Savić J., Marković N. (1994). In vitro assays for antitumor-drug screening on human tumor cell lines: Dye exclusion test and colorimetric cytotoxicity assay. Arch. Oncol..

[43] Higuchi K., Mitsuhashi N., Saitoh J., Maebayashi K., Sakurai H., Akimoto T., Niibe H. (2000). Caffeine enhanced radiosensitivity of rat tumor cells with a mutant-type p53 by inducing apoptosis in a p53-independent manner. Cancer Lett..

[44] Degtjarik O., Golovenko D., Diskin-Posner Y., Abrahmsen L., Rozenberg H., Shakked Z. (2021). Structural basis of reactivation of oncogenic p53 mutants by a small molecule: Methylene quinuclidinone (MQ). Nat. Commun..

[45] Wang Y., Rosengarth A., Luecke H. (2007). Structure of the human p53 core domain in the absence of DNA. Acta Crystallogr. D Biol. Crystallogr..

[46] Dassault Systèmes (2021). BIOVIA Discovery Studio.

[47] Morris G.M., Huey R., Lindstrom W., Sanner M.F., Belew R.K., Goodsell D.S., Olson A.J. (2009). AutoDock4 and AutoDockTools4: Automated docking with selective receptor flexibility. J. Comput. Chem..

[48] O’Boyle N.M., Banck M., James C.A., Morley C., Vandermeersch T., Hutchison G.R. (2011). Open Babel: An open chemical toolbox. J. Cheminform..

[49] Santos-Martins D., Solis-Vasquez L., Tillack A.F., Sanner M.F., Koch A., Forli S. (2021). Accelerating AutoDock4 with GPUs and gradient-based local search. J. Chem. Theory Comput..

[50] Mladenović T., Simijonović D., Rüffer T., Kaluđerović G.N., Milenković D., Jovičić Milić S., Avdović E., Marković Z. (2025). Green synthesis, structural characterization, BSA binding and NLO properties of newly synthesized pyrazol-chromeno [2,3-d] pyrimidine derivative: An experimental and theoretical study. J. Mol. Struct..

[51] Avdović E., Simijonović D., Milenković D., Mladenović T., Milutinović M., Nikodijević D., Cvetković D., Marković Z. (2026). Assessment of antioxidant activity, redox behavior, and human serum albumin binding of pyrogallol-coumarin hybrids. Results Chem..

[52] Chou T.C., Talalay P. (1984). Quantitative analysis of dose–effect relationships: The combined effects of multiple drugs or enzyme inhibitors. Adv. Enzym. Regul..

[53] Chou T.C. (2010). Drug combination studies and their synergy quantification using the Chou–Talalay method. Cancer Res..

