# Supplementary material for: Molecular Insights into the Interaction Between Metformin and Caffeine: The Time-Dependent Antagonism and Modulation of p53 Signaling in Cancer Cells
Source: Molecules. 2026 May 29;31(11):1877. doi: 10.3390/molecules31111877 (PMC13258596; doi:10.3390/molecules31111877)
Supplement: Supplementary file 1 [file molecules-31-01877-s001.zip › molecules-4266832-supplementary.pdf]

SUPPLEMENTARY MATERIAL
FOR

Molecular Insights into the Interaction between Metformin and Caffeine: Time-Dependent Antagonism and Modulation of p53 Signaling in Cancer Cells

Vesna Zeljković[1], Mirjana Bogavac[2], Milan Dekić[3], Slaviša Minić[1], Elvis Mahmutović[1], Vanja Kunkin[4], Zoran Marković[3] and Maja Karaman[5,*]

[1] Department of Biomedical Sciences, State University of Novi Pazar, Vuka Karadžića 9, 36300 Novi Pazar, Serbia; vzeljkovic@np.ac.rs (V.Z.); sminic@np.ac.rs (S.M.); ehmahmutovic@np.ac.rs (E.M.);

[2] Department of Obstetrics and Gynecology, Faculty of Medicine, University of Novi Sad, Hajduk Veljkova 3, 21000 Novi Sad, Serbia; mirjana.bogavac@mf.uns.ac.rs

[3] Department of Sciences and Mathematics, State University of Novi Pazar,VukaKaradžića9,
36300 Novi Pazar, Serbia; mdekic@np.ac.rs (M.D.); zmarkovic@uni.kg.ac.rs (Z.M.)

[4] Đorđe JoanovićZrenjanin General Hospital, Dr VaseSavića 5, 23000 Zrenjanin, Serbia; kunkinbre@gmail.com

[5] Department of Biology and Ecology, Faculty of Sciences, University of Novi Sad, Trg Dositeja Obradovića 2, 21000 Novi Sad, Serbia

* Correspondence: maja.karaman@dbe.uns.ac.rs

Table of Contents

Table S1. Standard Procedures for Calibration Curve Construction , https://www.abcam.com/

Table S2. Comparative Measurements in *HeLa* and *MRC*-5 Cells Following 24 h and 48 h of Incubation

Figure S1. Calibration curve for p53, https://www.abcam.com/

Figure S2. Molecular docking of metformin and caffeine to p53

Table S1. Standard Procedures for Calibration Curve Construction

Conc (U/ml)	O.D. 450nm
100	1.788
50	0.965
25	0.654
12.5	0.478
6.25	0.340
3.12	0.266
0	0.141

Table S2. Comparative Measurements in *HeLa* and *MRC*-5 Cells Following 24 h and 48 h of Incubation

24 h	Concentration

	Recorded (450nm)	(U/mL) *p53*	48 h Recorded (450nm)	Concentration (U/mL) p53
MRC-5	0.412	23.04	0.511	28.58
MRC-5 + Met[1]	0.385	21.53	0.415	23.21
MRC-5 + Caff[1]	0.365	20.41	0.395	22.09
HeLa	1.371	76.67	0.518	28.57
HeLa + Met	0.755	42.23	0.583	32.60
HeLa + Caff	0.572	31.99	0.464	25.95
Standard (S)	0.3	5.51	0.504	28.19
Control (C)	1.344	75.16	0.623	34.84

Footer[1]: Met – metformin, Caff – caffeine

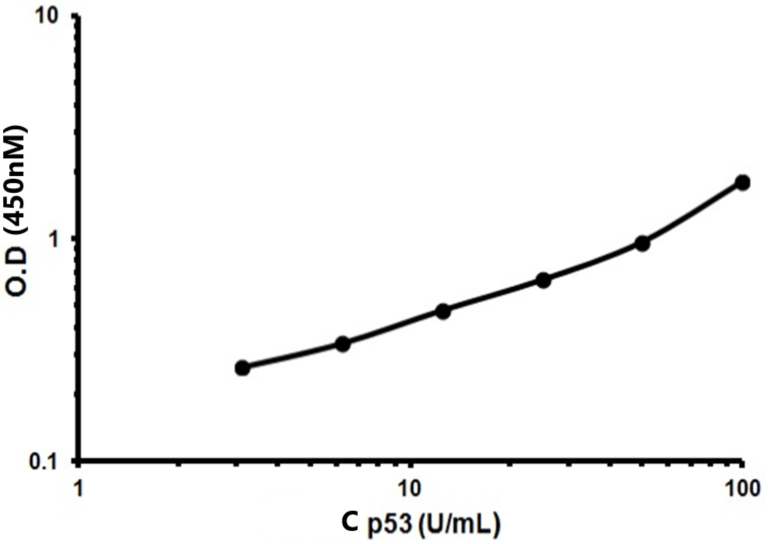

Figure S1. Calibration curve for p53

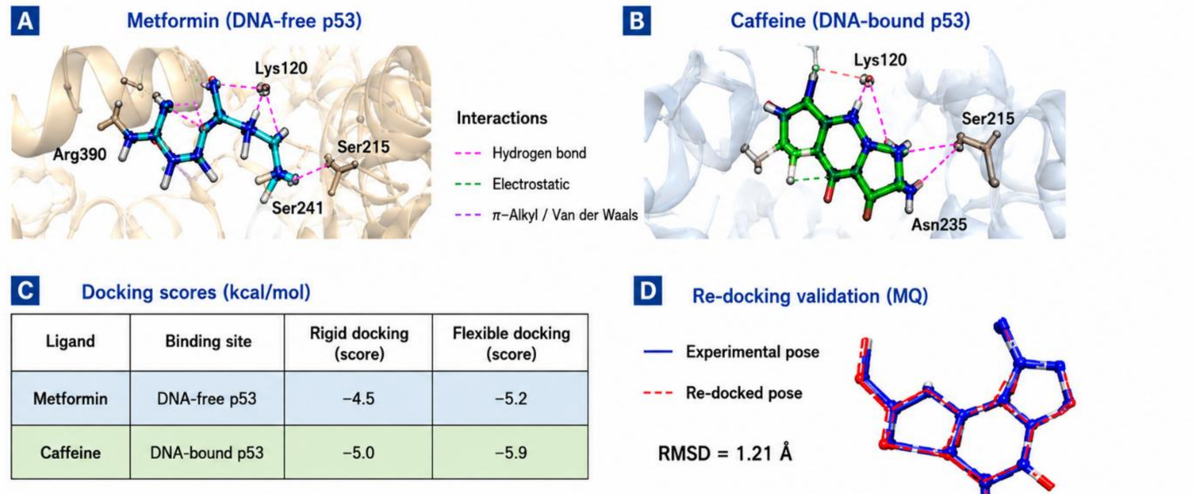

(A,B) Key interactions of metformin (A) and caffeine (B) within p53 binding sites.

(C) Best docking scores obtained by AutoDock Vina (rigid and flexible).

(D) Re-docking of methylene quinuclidinone (MQ) to p53 (PDB ID: 6IEE); RMSD = 1.21 Å.

Figure S2. Molecular docking of metformin and caffeine to p53
